# Seroprevalence of Anti-Chikungunya Virus Antibodies in Children and Adults in Managua, Nicaragua, After the First Chikungunya Epidemic, 2014-2015

**DOI:** 10.1371/journal.pntd.0004773

**Published:** 2016-06-20

**Authors:** Guillermina Kuan, Stephania Ramirez, Lionel Gresh, Sergio Ojeda, Marlon Melendez, Nery Sanchez, Damaris Collado, Nadezna Garcia, Juan Carlos Mercado, Aubree Gordon, Angel Balmaseda, Eva Harris

**Affiliations:** 1 Centro de Salud Sócrates Flores Vivas, Ministry of Health, Managua, Nicaragua; 2 Sustainable Sciences Institute, Managua, Nicaragua; 3 Division of Infectious Diseases and Vaccinology, School of Public Health, University of California, Berkeley, Berkeley, United States of America; 4 National Virology Laboratory, Centro Nacional de Diagnóstico y Referencia, Ministry of Health, Managua, Nicaragua; 5 Department of Epidemiology, School of Public Health, University of Michigan, Ann Arbor, United States of America; Florida Department of Health, UNITED STATES

## Abstract

Chikungunya is a viral disease transmitted by *Aedes aegypti* and *Ae*. *albopictus* mosquitoes. In late 2013, chikungunya virus (CHIKV) was introduced into the Caribbean island of St. Martin. Since then, approximately 2 million chikungunya cases have been reported by the Pan American Health Organization, and most countries in the Americas report autochthonous transmission of CHIKV. In Nicaragua, the first imported case was described in July 2014 and the first autochthonous case in September 2014. Here, we conducted two studies to analyze the seroprevalence of anti-CHIKV antibodies after the first chikungunya epidemic in a community-based cohort study (ages 2–14 years) and in a cross-sectional survey of persons aged ≥15 years in the same area of Managua, Nicaragua. Routine annual serum samples collected from 3,362 cohort participants in March/April 2014 and 2015, and 848 age-stratified samples collected from persons ≥15 years old at the end of May-beginning of June 2015 were used to estimate the seroprevalence of anti-CHIKV antibodies after the first epidemic (October 2014 to February 2015 in the study population). Using an Inhibition ELISA assay that measures total anti-CHIKV antibodies, the seroprevalence was significantly higher in those aged ≥15 (13.1% (95%CI: 10.9, 15.5)) than in the pediatric population (6.1% (95%CI: 5.3, 6.9)). The proportion of inapparent infections was 58.3% (95%CI: 51.5, 65.1) in children and 64.9% (95%CI: 55.2, 73.7) in the ≥15 study population. We identified age, water availability, household size, and socioeconomic status as factors associated with the presence of anti-CHIKV antibodies. Overall, this is the first report of CHIKV seropositivity in continental Latin America and provides useful information for public health authorities in the region.

## Author Summary

Chikungunya is a viral disease primarily characterized by high fever and joint pain. Chikungunya virus (CHIKV) is transmitted by infected *Aedes aegypti* and *Ae*. *albopictus* mosquitos. Although chikungunya was first described in 1952 and CHIKV has circulated in parts of Africa and Asia, since then, it was not introduced into the Americas until late 2013. Chikungunya poses a threat in tropical countries where the vector mosquitoes reside and in particular in CHIKV-naïve populations. In this study, we aimed to explore the dissemination of CHIKV through the first epidemic in Nicaragua and evaluate possible factors associated with infection. By analyzing the sera of two study populations, pediatric (2–14 years old) and ≥15 years participants, for anti-CHIKV antibodies, we determined who was infected during the first outbreak in Nicaragua. The seroprevalence of anti-CHIKV antibodies in the pediatric and ≥15 year-old study populations was 6.1% and 13.1%, respectively. Furthermore, using a demographic/household survey, we found that age, water availability, household size, and socioeconomic status were associated with CHIKV seropositivity. In conclusion, this study indicates the level of protective immunity the population has developed and can help government institutions develop intervention strategies.

## Introduction

Chikungunya virus (CHIKV) is an alphavirus belonging to the *Togaviridae* family and is primarily transmitted by *Aedes (Ae*.*) aegypti* and *Ae*. *albopictus* mosquitos [1]. The main epidemic cycle consists of human-mosquito-human transmission, although a natural reservoir of CHIKV in non-human primates serves as part of a sylvatic cycle in Africa, which maintains virus circulation during inter-epidemic periods [2, 3]. A bite from an infected mosquito transmits CHIKV, causing chikungunya, an acute viral illness characterized by high fever, arthalgia, myalgia and skin rash [4]. In the chronic stage of the disease, persistent or re-occurring arthralgia is common and may last for years [4, 5]. Historically, mortality due to chikungunya was thought to be unusual and only observed in the very young, old or immunocompromised; however, the during the outbreak in La Reunion Island in 2005–6, a case fatality rate of 1/1,000 was observed [6]. Even though the mortality rate remains in question, high attack rates are often seen throughout different epidemics [7].

Since the isolation of CHIKV after a 1952–1953 epidemic in present-day Tanzania [8], it has been endemic in parts of Africa and Asia; however, within the last decade it has reemerged as a major threat to human health globally, causing massive outbreaks in endemic areas, as well as in new regions [2]. After over 30 years of small, limited outbreaks, CHIKV resurfaced in Kenya and the Indian Ocean in 2004–5 [7, 9–11]. A combination of increased global travel and trade, wide distribution of the mosquito vectors, and lack of herd immunity contributes to the introduction and rapid spread of CHIKV in naïve populations. This was the case in parts of Europe, Asia and the Indian Ocean that reported locally transmitted cases of CHIKV for the first time [10, 12], as well as the recent introduction of CHIKV into the Americas [13].

In the Americas, autochthonous CHIKV transmission was first reported in the Caribbean on the island of St. Martin in December 2013 [13, 14]. Regional dissemination into Central/South/North America has affected more than 45 countries or regions, with approximately 2 million suspected cases, as reported by the Pan American Health Organization [15]. Nicaragua reported its first imported chikungunya case in July 2014 [16]. In Managua, the capital city, the first imported case was identified in August 2014 and the first locally transmitted case in September 2014 (A. Balmaseda, personal communication).

In this study, we performed a cross-sectional seroprevalence study of anti-CHIKV antibodies in District II of Managua in children aged 2–14 years enrolled in the Pediatric Dengue Cohort Study [17, 18] and in individuals aged ≥15 years recruited door-to-door specifically for this study. In both study populations, total anti-CHIKV antibodies were detected via Inhibition Enzyme Linked Immunosorbant Assay (ELISA), and factors associated with CHIKV seropositivity were evaluated using the same questionnaire. The objective of this study was to determine the seroprevalence of anti-CHIKV antibodies during the first chikungunya outbreak in Managua, correlate potential risk factors with CHIKV seropositivity, and show the spatial distribution of CHIKV seroprevalence in District II of Managua. Altogether, this study indicates the level of protective immunity the population has developed, identifies susceptible populations and factors associated with seropositivity, and can help government institutions develop intervention and mitigation strategies.

## Methods

### Ethics statement

The protocol for the Pediatric Dengue Cohort Study (PDCS) was reviewed and approved by the Institutional Review Boards (IRB) of the University of California, Berkeley, the University of Michigan, and the Nicaraguan Ministry of Health. Parents or legal guardians of all subjects provided written informed consent, and subjects 6 years of age and older provided oral assent. An amendment to add CHIKV infection screening to the PDCS, including specific consent/assent from the parent/guardian and the participant, was also approved by the IRBs reviewing the study. The protocol for the cross-sectional study of persons aged ≥15 years was reviewed and approved by the IRB of the Nicaraguan Ministry of Health and the Pan American Health Organization's Ethics Review Committee. Participants 18 years of age and older provided written informed consent. For participants aged 15–17, parents or legal guardians provided written informed consent and participants provided written assent.

### Study populations and sample collection

This study examined the seroprevalence of anti-CHIKV antibodies in two study populations, 2–14 years and ≥15 years of age, residing in the catchment area of the Health Center Sócrates Flores Vivas (HCSFV) in District II of Managua, Nicaragua. The total population served by HCSFV is ~62,000, with a population density of ~6,700 habitants/km^2^. The population aged 2–14 and ≥15 was estimated in 2015 at 14,240 and 45,312, respectively (G. Kuan, personal communication). The 15 neighborhoods of the study area are low- to mid-socioeconomic status; the illiteracy rate is 7%. The majority of homes, ranging from residential to shanty dwellings, are owner-occupied; close to 95% of the area has access to water and sewage infrastructure, and 88% has garbage collection services. Samples from participants 2–14 years old were obtained through the ongoing PDCS, originally established in 2004 to study dengue and dengue virus infections in Nicaragua [17, 18] and expanded to include CHIKV infection in children in September 2014 [16]. The PDCS consists of approximately 3,500 children aged 2–14 years, and children are provided with all of their primary health care through the study at the HCSFV. Acute and convalescent blood samples are collected from participants meeting the case definition of dengue and/or chikungunya or presenting with undifferentiated fever [16]. Additionally, a routine blood sample is collected annually in March/April from the children when they are healthy. During the annual sampling, the parents/guardians also complete a socioeconomic and household risk factor questionnaire. Participants who are sick when presenting for the annual sample collection, in particular those who had a fever within the previous week, are referred to a study physician and asked to return for sampling when healthy. Participants are enrolled and withdrawn throughout the year according to age criteria; thus, not all participants had routine annual samples collected in both 2014 and 2015. This study was restricted to those with paired 2014 and 2015 samples (n = 3,362, Table 1).

**Table 1:** Demographic data of pediatric and ≥15 year-old participants, District II of Managua, March–June 2015.

	Children (2–14 y/o)	≥15 y/o
**Participants—N**	3,362	848
**Households—N**	2,035	544
**Persons per household—mean (SD)**	8.1 (4.4)	7.5 (4.6)
**Female sex—N (%)**	1,685 (50.1)	614 (72.4)
**Age—mean (SD)**	7.9 (3.7)	43.8 (18.3)
**Age Groups (years)—N (%)**		
*2–4*	793 (23.6)	-
*5–9*	1,336 (39.7)	-
*10–14*	1,233 (36.7)	-
*15–29*	-	240 (28.3)
*30–44*	-	214 (25.2)
*45–59*	-	202 (23.8)
*≥ 60*	-	192 (22.6)
**Socioeconomic status^a^ - N (%)**		
*Not poor*	1,386 (41.2)	342 (40.3)
*Poor*	1,976 (58.8)	506 (59.7)
**Daily Hours Without Water—N (%)**		
*0*	2,432 (72.3)	637 (75.1)
*1–7*	486 (14.5)	73 (8.6)
*≥ 8*	444 (13.2)	138 (16.3)

^a^Socioeconomic status was evaluated using survey information on possessions, conditions of the home, and crowding provided by the participants or their parents/guardians.

Participants aged ≥15 were recruited as part of a community-based cross-sectional study conducted at the end of May/beginning of June 2015 in the same area as the cohort study. Age and residency were the only inclusion criteria. To be consistent with the PDCS, individuals who had experienced fever within the seven days prior to sampling were excluded from the study. To avoid clustering, a maximum of two people per household were allowed to participate. Sample size was estimated to determine the seroprevalence with a +/- 10% precision using the following parameters: estimated seroprevalence rate of 20%, type I error of 0.05, and power of 0.8. The estimated sample size was 502 participants. In order to adjust for clustering by household (enrollment of up to 2 persons per household), a correlation coefficient of 0.6 to 0.9 was used, yielding a final sample size of 604 to 904 participants. The study area in Managua was composed of 15 neighborhoods, and the sample size of each neighborhood was proportional to its total population. Each neighborhood is divided into blocks. The sample size of the neighborhood was distributed uniformly among blocks. Within a block, houses to be visited were randomly selected by study personnel. Within each household, potential participants were approached randomly. The target enrollment per household was 2 participants; however, in some households only one person consented to study participation. Approximately 150 individuals per ten-year age group (15–24, 25–34, 35–44, 45–54, 55–64, 65+) were sampled. Each participant was asked to complete a survey, which included information about the participant’s demographics, household, socioeconomic status and potential risk factors.

Surveillance of symptomatic chikungunya cases conducted in the cohort study indicated that, in this area of Managua, the first epidemic of chikungunya lasted from September 2014 until February 2015 [16], and the second epidemic began in June 2015 and lasted through February 2016. Therefore, by utilizing samples from pediatric and ≥15 year-old study populations, we were able to analyze the seroprevalence of anti-CHIKV antibodies during the first outbreak of chikungunya in Managua.

### Surveys

The same demographic and household survey was used in the two study populations. Demographic data collected included sex, age and education, and the household questionnaire included questions on appliance ownership and conditions of the home. In addition, participants aged ≥15 were asked to recall any self or clinical diagnosis of chikungunya (including symptoms and disease severity) and to describe their knowledge and attitudes towards chikungunya. In both studies, the survey was administered orally and answers were recorded by HCSFV interviewers on a mobile device (smart phone or tablet computer) using a custom-built application. Global positioning system (GPS) latitude and longitude coordinates were taken using the GPS capabilities of the mobile devices.

### Socio-economic status evaluation

To characterize the socio-economic status (SES) of each household, a wealth index was constructed using the following variables from the household survey: crowding (ratio of the number of people in the household divided by the number of bedrooms), number of fans, TVs, refrigerators, motor cycles, and/or cars owned, materials used to build the ceiling and the floor, and whether the family cooked with firewood. The wealth index was then analyzed using principal component analysis (PCA) [19]. PCA analyses were run separately for both study populations. As the number of households in the ≥15 years study population was relatively low, the households were divided into two halves, “poor” and “not poor”, according to their SES. To be consistent, the same approach was used for the pediatric study population. Individual participants were assigned the SES of their household.

### Inhibition ELISA

An in-house single-dilution inhibition Enzyme Linked Immunosorbent Assay (ELISA) was used to detect total CHIKV-specific antibodies in the serum of the participants (Saborio et al, submitted for publication). In this assay, CHIKV antigen produced in C6/36 cells is captured onto polystyrene plates using the anti-CHIKV monoclonal antibody (mAb) 187 (obtained from Dr. Michael S. Diamond, Washington University in St. Louis) [20]. CHIKV-specific antibodies that might be present in the participants’ samples then compete for binding to the CHIKV antigen with horseradish peroxidase (HRP)-conjugated anti-CHIKV mAb 152 (obtained from Dr. Michael S. Diamond) [20]. Using tetramethylbenzidine HRP substrate, bound mAb 152 is quantified by measuring the optical density (OD) at 450 nm on an ELISA reader. Samples with an OD lower than 50% of the negative controls' mean OD were considered seropositive. The sensitivity of this assay was measured using paired healthy annual samples collected in March/April 2014 and March/April 2015 in cohort participants who had experienced a laboratory-confirmed acute CHIKV infection in the period between the annual sample collections. The assay had a sensitivity of 92.4% (95%CI 86.9, 97.8), such that of the 92 chikungunya cases, 85 seroconverted (i.e., the sample was negative in 2014 and positive in 2015).

### Statistical analysis

Data were analyzed using STATA Software (STATA Corp, College Station TX). Relative and absolute frequencies are reported for categorical variables, and mean and standard deviation are reported for quantitative variables. A binomial distribution was used to calculate 95% confidence intervals (CIs) for seroprevalence and proportion of inapparent infections. The seroprevalence estimate was post-stratified using the 2015 age structure (G. Kuan, personal communication) and the 2005 gender structure [21] of the study area population. We used generalized linear models assuming a Poisson distribution in order to calculate prevalence ratios (PR). Crude and adjusted PR were calculated using univariate and multivariate analysis, respectively. Two multivariate models were constructed. One included all variables listed in Table 2 except the number of persons per household and one included all variables except SES. This was done as the number of persons per household was also used to determine the participant's SES. A mixed-effects Poisson regression was used to test the hypothesis that at least one neighborhood had a seroprevalence different to the seroprevalence of the entire study area. In the mixed-effects Poisson regression, the fixed effects were sex, age group, SES, and daily hours without water, and the random effect was the neighborhood. To identify neighborhoods with a seroprevalence different than the seroprevalence of the study area, random effects for the variable neighborhood and their 95%CIs were visually inspected for overlap (caterpillar plot).

**Table 2:** Prevalence and prevalence ratio of anti-CHIKV antibodies in participants aged 2–14 years, District II of Managua, March–April 2015.

	N	Prevalence (95%CI)	Prevalence ratio (95%CI)	Adjusted prevalence ratio (95%CI)
**Total**	3,362	6.1 (5.3, 6.9)	-	-
**Sex**				
*Female*	1,685	5.7 (4.6, 6.9)	1	1
*Male*	1,677	6.4 (5.3, 7.7)	1.13 (0.96, 1.32)	1.13 (0.97, 1.33)^b^
**Age (years)**				
*2–4*	793	4.8 (3.4, 6.5)	1	1
*5–9*	1,336	4.2 (3.2, 5.4)	0.87 (0.68, 1.10)	0.88 (0.69, 1.11)^b^
*10–14*	1,233	8.9 (7.4, 10.7)	**1.86 (1.50, 2.30)**	**1.85 (1.50, 2.29)^b^**
**Socioeconomic status^a^**				
*Not poor*	1,386	4.8 (3.8, 6.1)	1	1
*Poor*	1,976	6.9 (5.9, 8.1)	**1.43 (1.21, 1.69)**	**1.33 (1.11, 1.58)^b^**
**Daily Hours Without Water**				
*0*	2,432	5.8 (4.9, 6.8)	1	1
*1–7*	486	6.2 (4.2, 8.7)	1.07 (0.85, 1.34)	1.07 (0.85, 1.34)^b^
*≥ 8*	444	7.7 (5.4, 10.5)	**1.33 (1.06, 1.65)**	**1.31 (1.05, 1.63)^b^**
**Per additional person in the household**	-	-	**0.97 (0.95, 0.99)**	**0.96 (0.95, 0.98)^c^**

Estimates in bold are statistically significant (p<0.05).

^a^Socioeconomic status was evaluated using survey information on possessions, conditions of the home, and crowding provided by the participants or their parents/guardians.

^b^Estimates from a multivariate model including all variables except persons in the household.

^c^Estimate from a multivariate model including all variables except SES.

## Results

### Study population

A total of 3,362 paired annual samples from the pediatric cohort were collected in March/April 2014 and 2015 and analyzed in this study (Table 1). Of these, 1,685 (50.1%) were from females and 1,677 (49.9%) were from males, approximately a 1:1 ratio. Age was uniformly distributed in the pediatric cohort; one-year age groups 2 to 14 years old had in average 259 participants (range: 231–281). A total of 1,078 persons aged ≥15 were approached for participation in the community-based cross-sectional study. Of those, 848 (79.6%) consented to participate. The approval rate was significantly higher in females (84.1%) than in males (67.2%) (p<0.0001). This, combined with the fact that more women were at home when the study was conducted, resulted in female-to-male ratio of 2.6:1 among the 848 participants (Table 1). Participants were distributed across all ages, with the 15–29 year age group having the highest number of participants (240; 28.3%) and the 65+ group having the lowest number of participants (192, 22.6%).

The socioeconomic status (SES) distribution was similar in both studies; 58.8% and 59.7% of the participants lived in a household categorized as poor for participants aged 2–14 and ≥15, respectively (Table 1). The amount of time without water per household was determined based on self-reporting. A similar distribution was recorded among the households in both studies, with the majority of the households reporting access to water 24 hours per day (Table 1; 72.3% for pediatric and 75.1% for ≥15 year-old participants).

### Seroprevalence of anti-CHIKV antibodies

**Fig 1:**
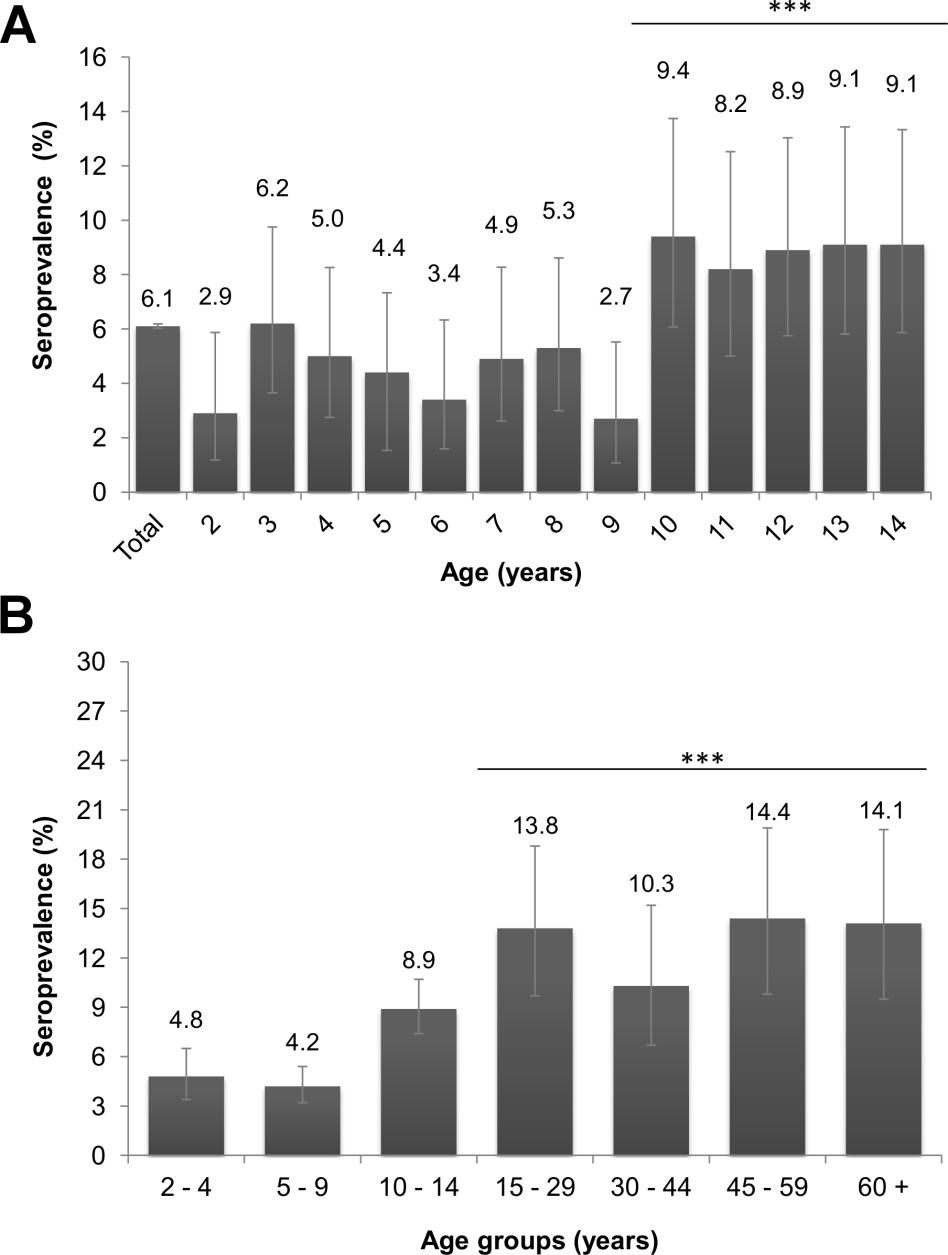
Seroprevalence of anti-CHIKV antibodies by age, District II of Managua, March–June 2015. **A. Pediatric population (2–14 years old).** The presence of anti-CHIKV antibodies in the serum/plasma of participants aged 2–14 years was determined via Inhibition ELISA. The seroprevalence is presented by year of age. There is a statistical difference between the participants <10 and ≥10 years old. ***P-value< 0.001. **B. Entire population (≥ 2 years old).** Presence of anti-CHIKV antibodies in the serum/plasma of both pediatric and ≥15 year-old participants was determined via Inhibition ELISA. Ages of pediatric participants were grouped into 2–4, 5–9 and 10–14 years old, while ≥15 year-old participants were divided into 15-year age groups. The seroprevalence of participants aged ≥15 is statistically higher when compared to the pediatric population. *** P-value<0.001.

The seropositivity in both study populations was determined via Inhibition ELISA. In the pediatric cohort study, paired samples were collected in March/April 2014, before the introduction of CHIKV into Nicaragua, and in March/April 2015, at the end of the first chikungunya epidemic and before the onset of the second epidemic, which began in mid-June 2015. As expected, no sample collected in 2014 was seropositive for anti-CHIKV antibodies (n = 3,362). In 2015, 204 children were seropositive for CHIKV, for a seroprevalence of 6.1% (95% Confidence Interval (CI): 5.3, 6.9) (Table 2). In the cross-sectional study of persons aged ≥15 years, single samples were collected at the end of May/beginning of June 2015. In this population, 13.1% (95%CI: 10.9, 15.4) of the 848 participants were seropositive for CHIKV (Table 3). Comparing the two populations, the seroprevalence in children aged 2–14 was 7.0 (95%CI: 4.6, 9.4) percentage points lower than those ≥15 (p<0.001) (Fig 1A). The post-stratified estimate of the seroprevalence for the population aged ≥2 years in the study area was 11.6% (95%CI: 9.7, 13.7).

**Table 3:** Prevalence and prevalence ratio of anti-CHIKV antibodies in participants aged ≥15 years, District II of Managua, May–June 2015.

	N	Prevalence (95%CI)	Prevalence ratio (95%CI)	Adjusted prevalence ratio (95%CI)
**Sex**				
*Female*	614	12.7 (10.2, 15.6)	1	1
*Male*	234	14.1 (9.9, 19.2)	1.11 (0.82, 1.49)	1.05 (0.78, 1.41)
**Age (years)**				
*15–29*	240	13.8 (9,7, 18.8)	1	1
*30–44*	214	10.3 (6.7, 15.2)	0.74 (0.50, 1.10)	0.70 (0.47, 1.04)
*45–59*	202	14.4 (9.8, 19.9)	1.04 (0.72, 1.50)	0.98 (0.68, 1.41)
*≥ 60*	192	14.1 (9.5, 19.8)	1.02 (0.70, 1.48)	0.93 (0.64, 1.36)
**Socioeconomic status^a^**				
*Not poor*	342	11.1 (7.9, 14.9)	1	1
*Poor*	506	14.4 (11.5, 17.8)	1.29 (0.97, 1.72)	1.11 (0.82, 1.51)
**Daily Hours Without Water**				
*0*	637	12.1 (9.7, 14.9)	1	1
*1–7*	73	17.8 (9.8, 28.5)	1.47 (0.95, 2.26)	1.48 (0.96, 2.28)
*≥ 8*	138	15.2 (9.7, 22.3)	1.25 (0.88, 1.78)	1.25 (0.88, 1.78)
**Per additional person in the household**	-	-	**0.94 (0.91, 0.98)**	**0.95 (0.92, 0.99)**

Estimates in bold are statistically significant (p<0.05).

^a^Socioeconomic status was evaluated using survey information on possessions, conditions of the home, and crowding provided by the participants or their parents/guardians.

### Proportion of CHIKV symptomatic and inapparent infection

In the pediatric cohort study, symptomatic chikungunya cases were captured through enhanced passive surveillance at the HCSFV since the introduction of CHIKV in the study population. Participants presenting with suspected chikungunya and/or dengue, as well as those with undifferentiated fever, were tested for acute CHIKV infection by real-time RT-PCR [16]. Participants positive for anti-CHIKV antibodies who did not present with an episode of laboratory-confirmed chikungunya were considered to have experienced an inapparent infection. In the study population aged ≥15, self-report was used to infer symptomatic and inapparent CHIKV infections. The proportion of inapparent infections among all participants with anti-CHIKV antibodies was 58.3% (95%CI: 51.5, 65.1) in the cohort population and 64.9% (95%CI: 55.2, 73.7) in the study population aged ≥15 years old.

### Effect of individual factors on chikungunya seroprevalence

Potential factors associated with CHIKV seropositivity were explored separately in both study populations. Gender was not associated with increased seroprevalence of anti-CHIKV antibodies in either population (Tables 2 and 3), but age was. In the pediatric population, there was a statistical association between increasing seropositivity and increasing age (Table 2). The oldest children in the cohort study, ages 10–14, had the highest seroprevalence of anti-CHIKV antibodies: 8.9% (95%CI: 7.4, 10.7). The adjusted prevalence ratio (aPR) for this age group compared to the 2–4 year-old group was 1.85 (95%CI: 1.50, 2.29). Seroprevalence was then calculated for each one-year age group (Fig 1B). Seroprevalence was consistently lower in one-year age groups from 2 to 9 years than in groups from 10 to 14. Accordingly, the aPR for children aged 10–14 was 2.00 (95%CI: 1.70, 2.35) when compared to those aged 2–9 (p<0.001). When analyzing only the participants aged ≥15, age was not demonstrated to be statistically associated with seropositivity (Table 3). However, when combining all participants in this study, participants with ages 2–14 displayed a lower seroprevalence than those ≥15 years old (p<0.001).

### Effect of household factors on seroprevalence

SES and household factors were evaluated as potential factors associated with seroprevalence of anti-CHIKV antibodies. Children characterized with a low SES (“poor"), based on the questionnaire parents/guardians answered, had a statistically higher seroprevalence (aPR: 1.33; 95%CI: 1.11, 1.58) than those in the higher SES “not poor” category (Table 2). Similarly, participants ≥15 years old with a low SES had a higher overall seroprevalence (aPR 1.11; 95%CI 0.82, 1.51); however, this difference was not statistically significant (Table 3). Another household factor that was examined was water availability. A statistically significant positive association between number of hours without water and anti-CHIKV seroprevalence was observed in pediatric study population, with the highest seroprevalence in the ≥8-hour group (Table 2); however, there was no significant association between hours without water and seroprevalence in the ≥15 year-old study population (Table 3). Household size (number of people per household) was statistically associated as a protective factor in both study populations (Tables 2 and 3).

### Spatial analysis

**Fig 2:**
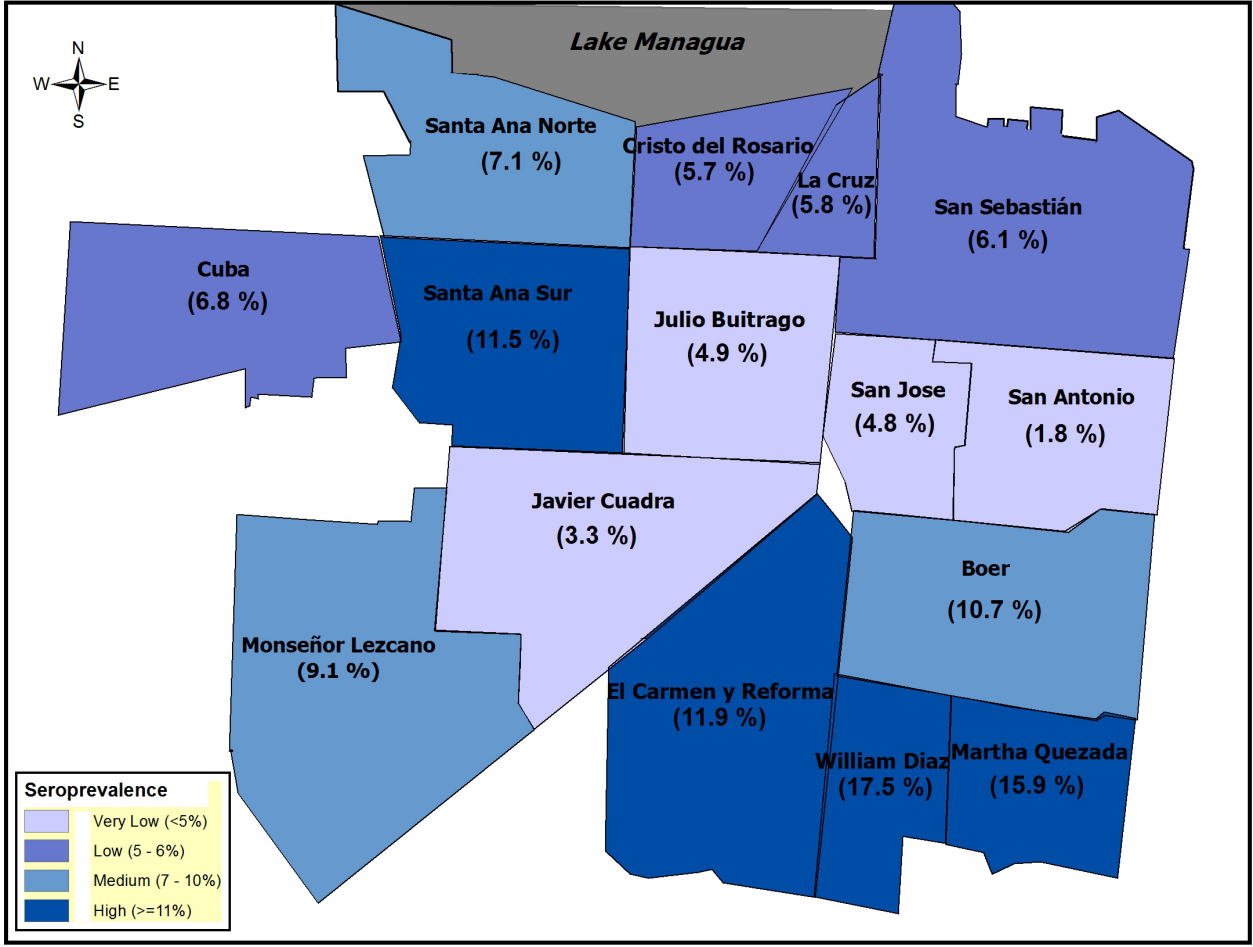
Seroprovalence of anti-CHIKV antibodies by neighborhood, District II of Managua, March–June 2015. The seroprevalence for each neighborhood was calculated using participants from both the pediatric (2–14 years) and ≥15 year-old study populations. Seroprevalence levels were divided into four groups and color-coded, as shown in the legend key.

Utilizing study household GPS coordinates, we visualized the spatial distribution of seroprevalence of anti-CHIKV antibodies by neighborhood. This study was conducted in a relatively small area of ~9 square kilometers. The seroprevalence in each neighborhood was calculated combining the participants from both studies. Seroprevalence varied 10-fold across neighborhoods: from 1.8% (95%CI: 0.05, 9.7) in San Antonio to 17.5% (95%CI: 7.3, 32.8) in William Díaz (Fig 2). Two neighborhoods had a seroprevalence significantly higher than the mean seroprevalence of the study area (p<0.001): Bóer and Santa Ana Sur. Moreover, 7 of the 15 neighborhoods consist of mixed commercial and residential areas, while the rest are mainly residential. Interestingly, 6 of 7 neighborhoods with commercial activity corresponded to the neighborhoods with the highest seroprevalence.

### Knowledge about chikungunya

The survey administered to ≥15 year-old participants included questions that addressed knowledge about chikungunya. The majority of the participants (93.5%) considered themselves informed about chikungunya (S1 Table). The major source of information was television (77.3%), followed by radio (11.7%). Other sources included community health workers, talks at the health centers/posts and newspapers. Knowledge about how chikungunya is transmitted was also evaluated. A total of 766 (90.3%) correctly identified mosquitoes as the disease vector. Participants were also asked about practices of chikungunya prevention implemented in their household. Of all participants, 758 (89.4%) implemented at least one measure (S1 Table). Knowledge or practices did not impact seroprevalence rates (S1 Table).

## Discussion

This study is the first to report seroprevalence and factors associated with CHIKV seropositivity in continental Latin America. The seroprevalence of anti-CHIKV antibodies were measured in two studies conducted in the same area of Managua, Nicaragua: an ongoing pediatric (2–14 years old) cohort study [17, 18] and a cross-sectional study in participants aged ≥15 years. The seroprevalence in the pediatric study was 6.1% (95%CI: 5.3, 6.9), while it was 13.1% (95%CI: 10.9, 15.5) in the ≥15 year-old study population. Moreover, older children had a higher seroprevalence than younger children. Increased number of hours without water in the household, lower socioeconomic status and lower number of people per household resulted in an increased risk for CHIKV infection. The proportion of inapparent infections among all infections was 58.3% (95%CI: 51.5, 65.1) in children and 64.9% (95%CI: 55.2, 73.7) in participants ≥15 years. The seroprevalence within the study area varied spatially, with neighborhood seroprevalence ranging from 1.8 to 17.5%.

In comparison to other seroprevalence studies, the seropositivity in our study is similar to Northern Italy, 10.2% [22], and St. Martin’s Island, 16.9% [23]. However, other studies performed in Asia, Africa and the Indian Ocean report higher seroprevalence, ranging from 25% to 75% [24–29]. The differences in seroprevalence between studies could be due to multiple factors. First, the strength of the outbreak/epidemic, indicated by the number of cases reported, could differ. Also, studies that measure anti-CHIKV IgG antibodies in endemic regions are likely detecting cumulative anti-CHIKV antibodies from previous CHIKV outbreaks [25, 28], whereas this study reflects the first introduction of CHIKV into Nicaragua. Mosquito distribution differs in different countries, and transmission of CHIKV can be affected by the vector species, due to adaptive mutations in CHIKV that expand its vector competence [30]. Furthermore, some studies [10, 24, 26] were conducted in areas where circulating CHIKV strains were different from the Asian genotype strains identified in Nicaragua [31]. Strains imported into Nicaragua in August 2015 had *E1* gene sequences that were 100% identical to the first strain sequenced in the Americas, BritishVirginIslands/99659/KJ451624/2014 [31].

Although no seroprevalence studies of anti-CHIKV antibodies in continental Latin America have been published to date, clinical attack rates in certain countries during the 2014–2015 epidemic were reported as very high (e.g., up to 81% in some areas of the Dominican Republic) [32, 33]. Several factors could contribute to the lower rate in Nicaragua. First, the country experienced a drought in 2014 that resulted in a delayed rainy season with less rainfall [34], which could have reduced entomological indices. Notably, Breteau indices in the study area were lower in 2014 than in 2011–2013 (G. Kuan, personal communication). However, the low seroprevalence after the first chikungunya epidemic indicates that the majority of the population remains susceptible to CHIKV infection and herd immunity has not yet been achieved. Consistent with this, the second chikungunya epidemic, which began in June 2015 and lasted until February 2016, was much larger than the first epidemic described in this report (September 2014 to February 2015). We plan on documenting the increase in seroprevalence by examining the same pediatric and ≥15 year-old participants in March-May of 2016.

The proportion of inapparent infections observed in this study was high, 58.3% (95%CI: 51.5, 65.1) in children and 64.9% (95%CI: 55.2, 73.7) in ≥15 year-old participants. The majority of seroprevalence studies have been conducted in areas where the East/Central/South African (ECSA) genotype of CHIKV had circulated [2]. These studies report proportions of inapparent infections ranging from 3.8% to 47.1% [10, 22–28, 35] and only two report proportions higher than 40% [28, 35]. Two studies were carried out in areas where the same CHIKV genotype as the one present in Nicaragua circulated but outside of the Americas: a small study conducted in Malaysia in 2007 (17.5% inapparent infections) [25] and a cohort study in the Philippines in 2012–2013 (82.1% inapparent infections) [36]. Finally, a study conducted on the island of St. Martin, the point of introduction of CHIKV in the Americas [23], recently reported a proportion of inapparent infections of 39.0%. Taken together, these studies and this report appear to point towards a higher proportion of inapparent CHIKV infections during outbreaks/epidemics caused by the Asian genotype.

In this study, factors associated with CHIKV seropositivity were age, water availability, household size, and SES. Seroprevalence increased with age in the pediatric population. Moreover, ≥15 years old participants had a seroprevalence of anti-CHIKV antibodies two times higher than participants aged 2–14. This observation is consistent with other outbreaks, which report lower seroprevalence in children [22, 24, 25, 27, 28]. In this study, gender was not associated with CHIKV seropositivity. Some previous seroprevalence studies report women having a higher seroprevalence [26, 29], while others report the contrary [22, 24, 27], and one study reports no gender difference [28].

Socioeconomic level was analyzed in this study using a wealth index. Lower SES correlated with increased CHIKV seropositivity. However, this increase was statistically significant only in the 2–14 year-old population, likely as a result of the larger sample size in this population compared to the ≥15-year-old study population. Similarly, an increase in the number of hours without water supply was correlated with an increased seroprevalence, but only reached significance in the 2–14 years population. Water availability can be a potential risk factor associated with CHIKV seroprevalence since *Ae*. *aegypti* mosquitoes that transmit the virus breed in clean water in and around people’s homes. We hypothesize that a greater number of hours without water represents a higher chance that the household will store water, thus increasing the number and volume of mosquito breeding containers. In contrast, household size was a protective factor in both study populations. A higher number of people in a household might decrease the likelihood of each individual getting bitten by an infected mosquito, and a lower vector-to-person ratio could be protective at the individual level. Although this study was conducted in a limited geographic area, seroprevalence varied 10-fold across neighborhoods. As reported in a seroprevalence study conducted in Bagan Pashor, Malaysia [25], neighborhoods with higher commercial activity in Managua showed higher seroprevalence.

This study has certain limitations. Both the pediatric and ≥15 year-old seroprevalence levels were lower than expected, and because of this, the sample size for the ≥15 study was underestimated. As a result, some factors demonstrated trends, rather than statistically significant associations. The majority of the people available during recruitment of the ≥15 year-old study population were females because men in the study area are less likely to be at home during the day. In addition, males who were approached were less likely to participate in the study than females. Another point is that symptomatic cases in the ≥15-year-old study population were self-reported, which might bias the estimate of the proportion of symptomatic infections in this population. However, the proportion of inapparent infections in the ≥15 year-old study population (64.9%) was quite similar to that in children (58.3%), where symptomatic cases were laboratory-confirmed acute cases in the cohort study [16]. Finally, the Inhibition ELISA used to detect anti-CHIKV antibodies had a sensitivity of 92.4%. Given the 95% CI of the assay sensitivity, the seroprevalence can be estimated at 6.2–7.0% and 13.4–15.1% in the pediatric and ≥15 year-old study populations, respectively.

Overall, we report seroprevalence of anti-CHIKV antibodies after the first epidemic in Managua, Nicaragua, and analyze factors associated with CHIKV seropositivity. In this newly exposed population, a low seroprevalence was documented, and we expected (and observed) an elevated seroprevalence in the second epidemic because the vast majority of the population was still susceptible after the first chikungunya epidemic. These data help the Ministry of Health in Nicaragua plan appropriate vector control and new mitigation strategies to confront the current CHIKV epidemics and provide useful information about the epidemic of chikungunya in the Americas.

## Supporting Information

S1 Checklist STROBE Checklist for cohort studies. (PDF)

S1 Table Knowledge and practices about chikungunya in the ≥15 years study population. (PDF)
