# Supplementary material for: Seroprevalence of Anti-Chikungunya Virus Antibodies in Children and Adults in Managua, Nicaragua, After the First Chikungunya Epidemic, 2014-2015
Source: PLoS Negl Trop Dis. 2016 Jun 20;10(6):e0004773. doi: 10.1371/journal.pntd.0004773 (PMC4913910; doi:10.1371/journal.pntd.0004773)
Supplement: S1 Table — (PDF) [file pntd.0004773.s002.pdf]

Table S1. Knowledge and practices about chikungunya in the ≥15 years study population

	N (%)	Seroprevalence (95%CI)
**Considers him/herself informed about chikungunya**		
Yes	793 (93.5)	13.2 (10.9, 15.6)
No	55 (6.5)	10.9 (2.4, 19.4)
**Main source of information about chikungunya**		
TV	613 (77.3)	
Radio	93 (11.7)	
Community health workers	45 (5.7)	
Talks at health center/post	23 (2.9)	
Newspapers	16 (2.0)	
Other	3 (0.4)	
**Correctly identifies mosquitoes as vector of transmission**		
Yes	766 (90.3)	13.6 (11.2, 16.2)
No	82 (9.7)	8.5 (3.5, 16.8)
**Implements at least one measure of chikungunya control**		
Yes	758 (95.4)	13.3 (10.9, 15.9)
No	90 (10.6)	11.1 (5.5, 19.5)
**Practices of chikungunya prevention***		
Elimination of mosquito breeding sites	620 (73.1)	
Covering water containers	482 (56.8)	
Mosquito nets	411 (48.5)	
Repellent	374 (44.1)	
Cleaning water containers	325 (38.3)	
Window screens	159 (18.8)	

* Multiple choices possible.
